# Over-expressing NadA quinolinate synthase in *Escherichia coli* enhances the bioelectrochemistry in microbial fuel cells

**DOI:** 10.1242/bio.059554

**Published:** 2023-03-13

**Authors:** Zhenyu Guo, Lei Wang, Changyuan Yu

**Affiliations:** Department of Pharmaceutical Engineering, College of Life Sciences and Technology, Beijing University of Chemical Technology, Beijing 100029, China

**Keywords:** Microbial fuel cell, Electrochemically active bacteria, Nicotinamide adenine dinucleotide A, NAD (H/^+^)

## Abstract

The microbial fuel cell (MFC), which converts biomass energy into electricity through microbial metabolism, is one of the important devices for generating new bioenergy. However, low power production efficiency limits the development of MFCs. One possible method to solve this problem is to genetically modify the microbial metabolism pathways to enhance the efficiency of MFCs. In this study, we over-expressed the nicotinamide adenine dinucleotide A quinolinate synthase gene (*nadA*) in order to increase the NADH/^+^ level in *Escherichia coli* and obtain a new electrochemically active bacteria strain. The following experiments showed an enhanced performance of the MFC, including increased peak voltage output (70.81 mV) and power density (0.29 μW/cm^2^), which increased by 361% and 20.83% compared to the control group, respectively. These data suggest that genetic modification of electricity producing microbes could be a potential way to improve MFC performance.

## Teaser

**Summary:** This article is a report on exploring the application of gene modification in microbial fuel cells (MFCs), which expands the study of gene modification in improving MFC performance.

## INTRODUCTION

The microbial fuel cell (MFC) is a bioelectrochemical reactor that generates electricity from organic substrates using electricity-producing microorganisms (Hampton et al., 2021; Grzegorz et al., 2019). It attracted a lot of attention because of its potential applications in dealing with the energy crisis and sewage treatment (Liu et al., 2020; Li et al., 2018; Uwe et al., 2003; Yousefi et al., 2021). However, low power generating efficiency is still a key bottleneck that needs to be addressed (Zhang et al., 2017; Bruce, 2009). It has been indicated that power generation can be improved by optimizing cell structures, the development of electrode materials, and most fundamentally enhancing the electricity-producing ability of microorganisms (Ren, 2017).

Screening of newly identified microorganisms, such as electrochemically active bacteria (EAB), is a widely used method for researchers (Iskander et al., 2017; Liu and Yin, 2017). However, genetic modification of EAB to enhance bioelectricity generation would be more feasible. Both the increased number of releasable electrons and the improved efficiency of transferring released electrons to the anodes have been described previously (Lital, 2010; Luo et al., 2018). Through random transposon insertion mutations in *S. oneidensis* MR-1 which promoted bioanabolism of cell-surface polysaccharides, Kouzuma et al. generated approximately 50% more current than using the wild-type strain (Kouzuma et al., 2010). Bretschger et al. made a series of deletion mutants in *mtrA*, *mtrB*, *mtrC*, *gspD*, *gspG*, etc., and studied consequent effects in MFCs (Bretschger et al., 2007). Berríos-Rivera et al. reported that over-expression of *pncB* elevated the total NAD levels (Berríos-Rivera et al., 2002a,b). These findings also suggest that enhanced metabolic pathway or ATP synthesis contribute to the electricity-producing efficiency of EAB.

Cofactors play crucial roles in regulating energy metabolism. Nicotinamide adenine dinucleotide (NAD^+^), with its reduced form NADH, are essential cofactors as electron carriers involved in cellular metabolic process and energy production (Minhas et al., 2019; Berríos-Rivera et al., 2002a,b). Accelerating anabolism of NAD by over-expressing the Nad quinolinate synthase (NadA) could enhance the bioelectrical properties of EAB (Belenky et al., 2007). NadA has recently been cloned and could regulate the NAD biosynthesis process indirectly by catalyzing the depletion of L-aspartic acid (Akira et al., 2006; Liang et al., 2019). As a result, we supposed that increased expression of *nadA* could induce an enlarged NAD (H/^+^) pool and consequently enhance the electricity production of EAB.

In this study, we constructed a plasmid vector bearing the wild-type *nadA* gene from *Escherichia coli*. Over-expression of *nadA* in *E. coli* BL21 (DE3) strongly increased the voltage in regular MFC devices with aspartic acid as the substrate. Our study provides evidence for an effective approach through genetic modification of EAB to enhance the electricity producing ability of MFCs.

## RESULTS

### Over-expressing *nadA* in *E. coli*

The 1044-bp wild-type *nadA* gene was cloned from *E. coli* BL21 using polymerase chain reaction (PCR) and then we followed routine genetic techniques for construction of over-expressing plasmids. Sequencing analysis proved the consistency during the vector recombinant process (data now shown). Recombinant plasmid vectors bearing the wild-type *nadA* gene were transformed into *E. coli* BL21.

**Fig. 1.**
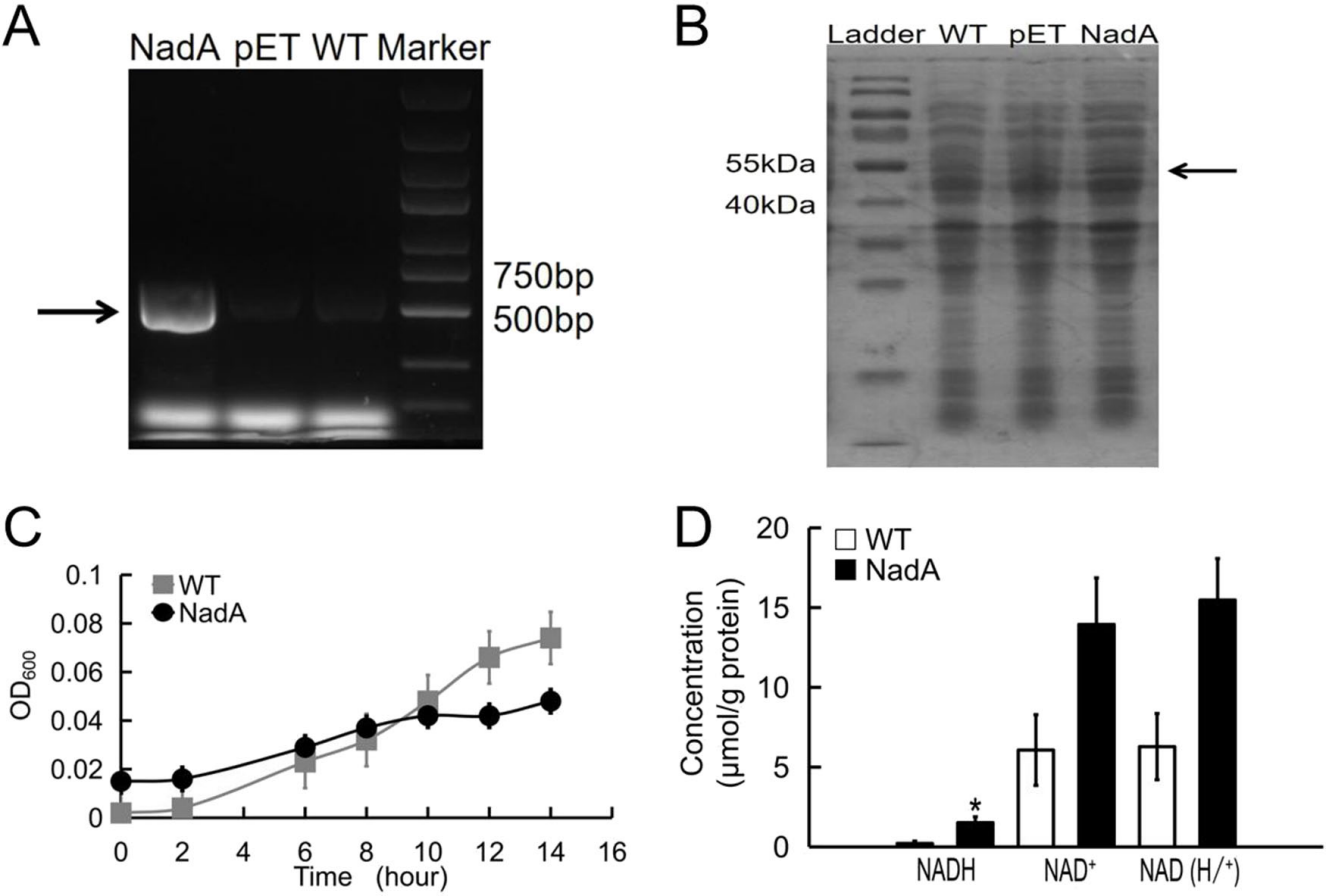
**Expression of *nadA* gene in *E. coli* after IPTG induction.** NadA mRNA levels were detected by RT-PCR (A). Increased protein concentration of NadA was shown after gel electrophoresis (B). The growth curves of bacterial within 14 h in LB medium. *n*=3 (C). Increased NAD (H/^+^) contents after *nadA* over-expression. *n*=3 (D). *t*-test, *P<0.05. WT, wild type; pET, blank vector control.

Gene expression was induced with 50 mg/ml IPTG at 37°C for about 8 h. Bacteria were collected, then the total mRNA and proteins were extracted, respectively. The mRNA level of *nadA* was detected through RT-PCR. A significant increase in *nadA* cDNA concentration was found compared with bacteria transformed with blank vectors (Fig. 1A). In addition, an increased *nadA* protein level was shown after gel electrophoresis (SDS-PAGE) following Coomassie Brilliant Blue staining (thick band indicated with an arrow in Fig. 1B).

Because over-expression of exogeneous proteins may cause a burden on the growth status, we detected the OD_600_ of cultured medium with bacteria. Over-expressing NadA protein slightly suppressed the amplification of *E. coli* in Luria-Bertani (LB) medium within 14 h (Fig. 1C).

### Effects of increased NadA on intracellular NAD (H/^+^)

As described previously (Liao et al., 2019), *nadA* functions in a salvage pathway and catalyzed the synthesis of NAD. An increased NAD (H/^+^) pool may enhance the metabolism of aspartic acid in EAB. We next analyzed the aspartic acid concentrations in anodic chambers with the WST-8 method after 12 days incubation. As Fig. 1D shows, the normalized NADH content was clearly augmented (*P=0.03). The NAD^+^ and NAD (H/^+^) pool also increased but were not statistically significant (*P*-values were 0.1 and 0.05, respectively).

### Over-expressing *nadA* in *E. coli* enhanced the performance of MFCs

We hypothesized that over-expressing *nadA* could increase the NAD (H/^+^) pool. As a result, the electron carrier would be increased, which could facilitate energy metabolism. In order to evaluate the electricity producing efficiency, we set up MFC devices and cultured *nadA* over-expressed *E. coli* in the anodic chambers. After a 2-week incubation, freshly prepared medium was added, and the voltage outputs were collected for about 2 weeks until the peak values were obtained.

**Fig. 2.**
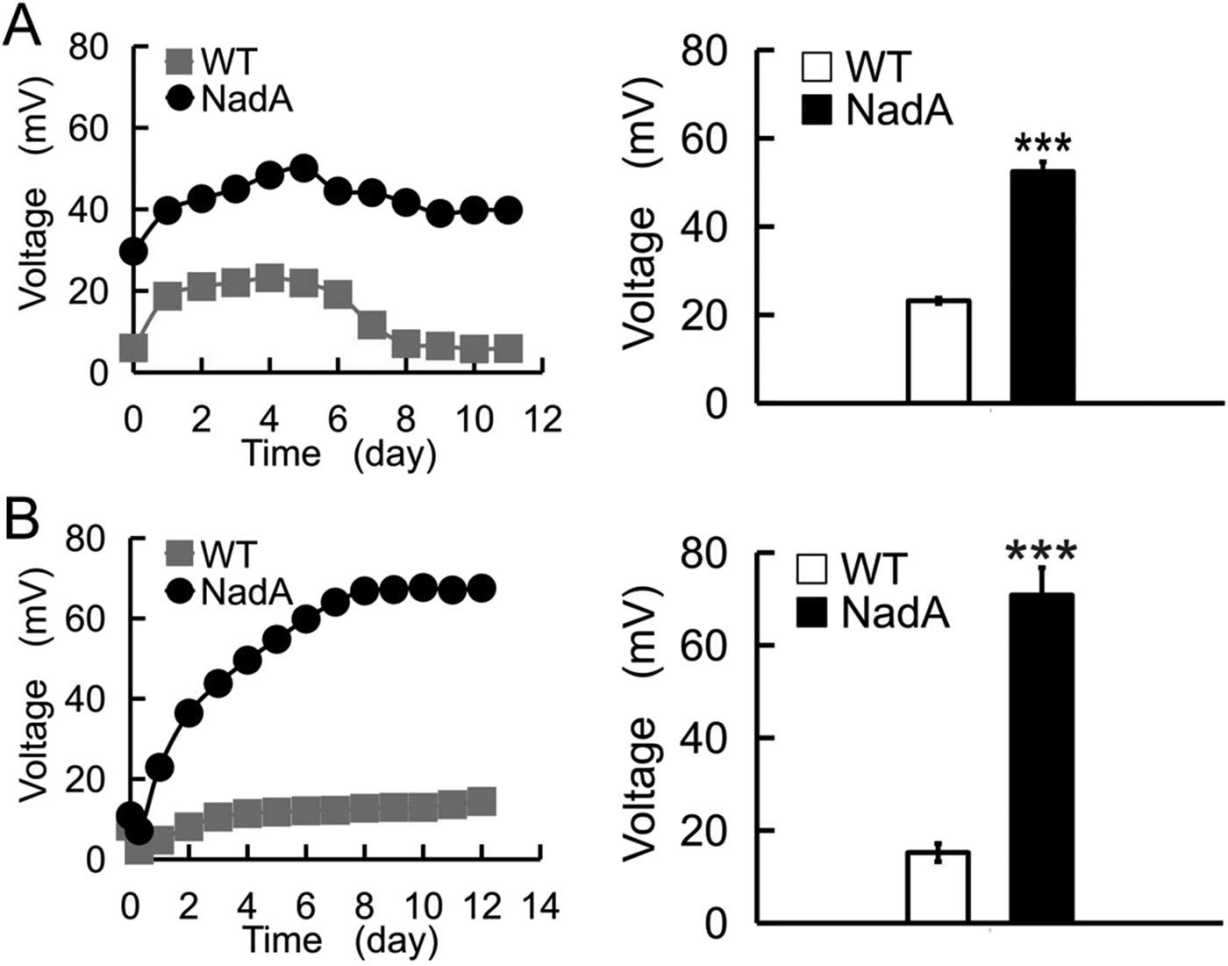
**Over-expressing *nadA* enhanced the performance of MFC.** The voltage output curves and the peak values were plotted for MFC with 1 g/L (A) and 5 g/L (B) aspartic acid as the substrate, respectively. *n*=3.

In *E*. *coli*, aspartic acid can be used as the substrate to synthesize NAD (H/^+^), and to generate energy through the tricarboxylic acid cycle (TCA). First, we used 1 g/l aspartic acid as the substrate following a previous report (Nishimura and Kisumi, 1984). Compared with the wild-type strain, which only produced limited voltage output (around 20 mV), *E*. *coli* with increased *nadA* levels significantly enhanced the electricity producing efficiency as indicated in Fig. 2. We plotted the voltage output curves and peak column diagram (Fig. 2A) during these days. There was a more than two times (52.46 mV) increase compared with the control group. Next, we tried a higher concentration of aspartic acid (5 g/l) to see whether the performance of the MFCs would be better. Indeed, we found a more than three times elevation of the peak voltage output (70.81 mV) (Fig. 2B).

**Fig. 3.**
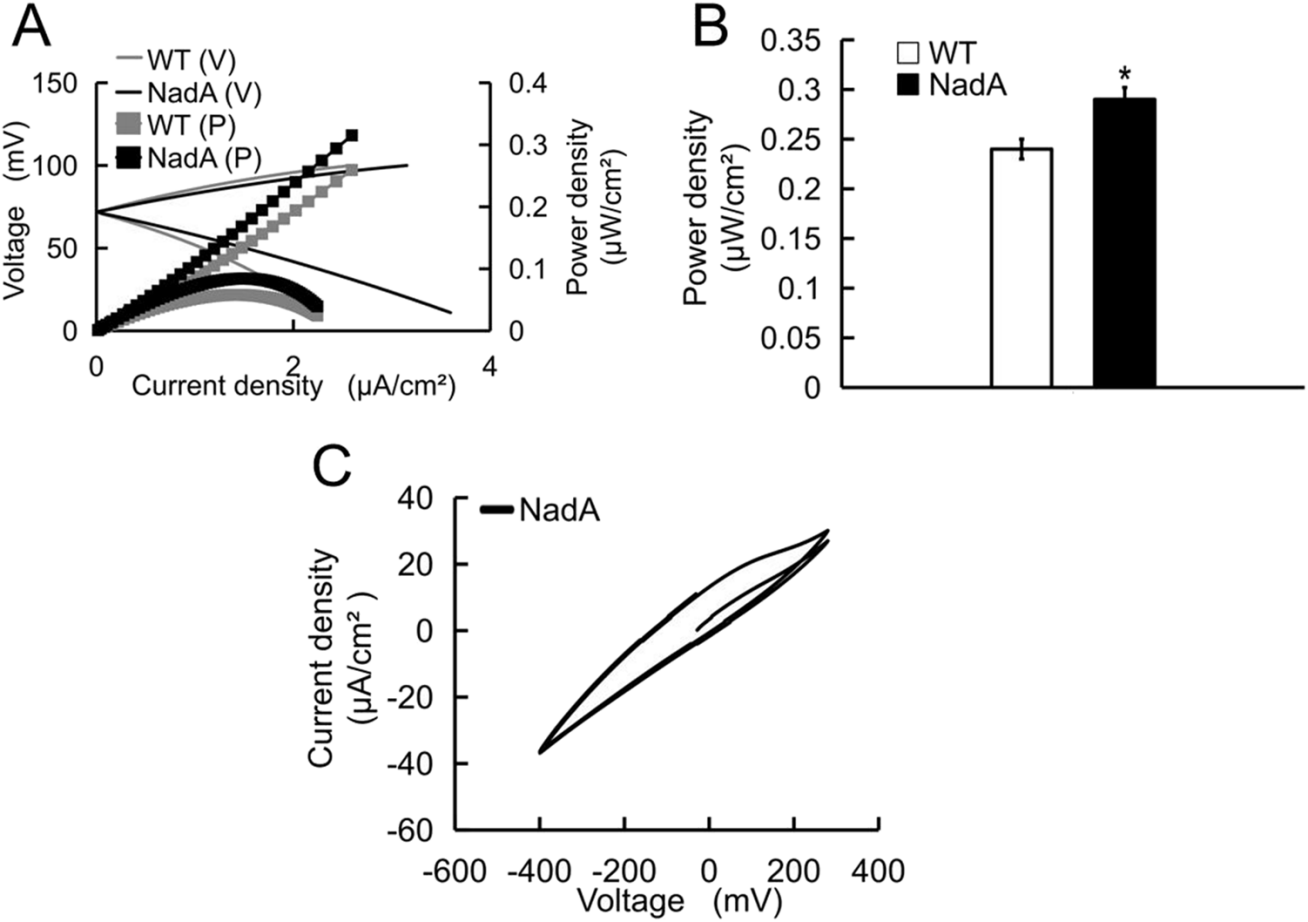
**Electrochemical performance of MFCs.** Power density curves (A) and power density column diagram (B) were plotted after scanning with electrochemical workstation. Two cycles recording of the voltage-current curves of the NadA group (C). *n*=3.

To better analyze the overall performance of MFCs, we then recorded the electrochemical performance with an electrochemical workstation. As shown in Fig. 3A and B, the peak value of power density was 0.29 μW/cm^2^ for NadA group, which was 20.83% higher than that of the control group (0.24 μW/cm^2^). In Fig. 3C, the voltage-current curves from two cycles of recording were overlaped, indicating a relatively stable electrochemical performance of the NadA group.

## DISCUSSION

With the hypothesis that increased expression of the cofactor NAD^+^/NADH could enhanced the electricity producing ability of EAB, we over-expressed *nadA* gene in *E. coli* BL21 (DE3). We found an increased concentration of NAD^+^ and slightly increased NADH. After setting up the MFC devices, we recorded remarkably enhanced peak voltage output in parallel with over-expression of *nadA*. In addition, the power density was increased accordingly.

Multiple strategies were tested to improve the performance of MFCs. Elevating the EAB's metabolism was proved to be effective (Schröder, 2007; Förster et al., 2003). It strengthened coulombic efficiency of MFCs with energy efficiency of 77.6% based on electron transfer. Meanwhile, over-expression of *nadA* gene in *E. coli* may promote the stability of Fe-S cluster (Rousset et al., 2008; Chan et al., 2012) thus enhancing the electrochemical activity of genetically modified strain with a steady increase in NADH. Findings in this work were consistent with a previous report that increasing the concentration of cofactors in *Pseudomonas aeruginosa*, such as NAD^+^/NADH, could enhance the electricity production of MFCs (Yong et al., 2014). Our data further suggested the effectiveness of genetic modification of metabolic pathway in EAB to enhance the electricity producing performance.

## MATERIALS AND METHODS

### Device

Two-chamber MFC devices were assembled following reported papers (Liu et al., 2006; Arkatkar et al., 2021). Anodic and cathodic chambers were separated by proton exchange membrane (Dupont, Beijing, China). The volume of each chamber was 100 cm^3^. Carbon clothes (5 cm×5 cm) were utilized in both the anodic and cathodic chambers. All MFC devices were performed at room temperature.

Anode buffer: L-aspartic acid (1, 5 g/l), ammonium chloride (0.19 g/l), sodium chloride (0.5 g/l), calcium chloride dihydrate (0.03 g/l), magnesium sulfate heptahydrate (0.03 g/l), sodium hydrogen carbonate (1 g/l), potassium phosphate monobasic (5 g/l), potassium phosphate dibasic (3.86 g/l), trace elements (12.5 ml/l).

Cathode buffer: sodium chloride (0.5 g/l), calcium chloride dihydrate (0.03 g/l), magnesium sulfate heptahydrate (0.03 g/l), sodium hydrogen carbonate (1 g/L), potassium phosphate monobasic (5 g/l), potassium phosphate dibasic (3.86 g/l), sodium nitrate (0.3 g/l), trace elements (12.5 ml/l).

Trace elements: nitrilotriacetic acid (1.5 g/l), magnesium sulfate heptahydrate (6.14 g/l), manganous sulfate (0.5 g/l), sodium chloride (1 g/l), iron (II) sulfate heptahydrate (0.1 g/l), calcium chloride dihydrate (0.1 g/l), cobaltous chloride (0.1 g/l), zinc chloride (0.13 g/l), copper (II) sulfate pentahydrate (0.01 g/l), aluminium potassium sulfate dodecahydrate (0.01 g/l), boric acid (0.01 g/l), sodium molybdate (0.03 g/l).

### Bacterial strains and plasmids

The *E. coli* series Top10 and BL21 (DE3) were purchased from Tiangen Co., Ltd (Beijing, China). pET-28 (a+) plasmid vector was a lab stock. Primers (nadA Primer 1:5′-CGCGGATCCATGAGCGTAATGTTTGATCCAGACA-3′; nadA Primer 2: 5′-CCGTCTCGAGTCCACGTAGTGTAGCCGCAAAA-3′; cDNA upstream: 5′-ATGGCGCGCTTCGGTGCAAA-3′; cDNA downstream: 5′-CTCTGATGTGGCAATGTTTTCGCAGCAGC-3′) were preserved in −20°C. All strains and plasmids were preserved in −80°C. *E. coli* were cultured with routine procedures at 37°C in LB medium.

The influences on growth status of *E. coli* by over-expressing *nadA* was indicated by the cell concentrations, which were detected by measuring the OD_600_ values with ultraviolet spectrophotometry. The strains were cultured oscillatory with 220 rpm at 37°C in LB medium for about 14 h.

### Quantification of intracellular NAD (H/^+^)

NAD (H/^+^) content was examined following the instruction of the determination kit. Briefly, the NAD (H/^+^) and the protein samples were extracted from bacterial after 5 h. The protein contents were determined with BCA protein standard curve (BCA Protein Assay Kit, Solarbio, Beijing, China). The NAD (H/^+^) concentrations were detected with NAD^+^/NADH Assay Kit (Beyotime, Beijing, China) and then normalized by the protein concentrations.

### Analysis of MFC performance

*E. coli* strains were imported into MFCs. Voltage outputs were collected for about 12 days by an EM9636 voltage collector (Zhongtai, Beijing, China) at a particular resistance (1000 Ω) connected to MFC device in different conditions (1 g/l or 5 g/l aspartic acid) (Nishimura and Kisumi, 1984). The plotted voltage output curves and averaged output peaks were used to analyze the electricity producing efficiency.

Power density and voltage-current curve were measured following the published paper. The 7-day power density (5 g/l aspartic acid) was recorded by a CH1660E electrochemical workstation (Chenhua, Shanghai, China) with scanning parameters were set as init E (0.1 V), final E (0.01 V), scan rate (0.01 V/s), run time (30 s), sensitivity (1×10^−4^ A/V). Scanning parameters for voltage-current curve were init E (0.28 V), final E (0.05 V), scan rate (0.01 V/s), run time (60 s) and two cycles.

### Statistical analysis

Data were averaged from three independent experiments.

Results were expressed as the mean±s.e.m. *t*-test with SPSS 19.0 software was used to perform the statistical analysis. *P<0.05, **P<0.01, and ***P<0.001.
